# Local Anesthetic Systemic Toxicity in an Infant Following a Caudal Block: A Case Report and Review of Literature

**DOI:** 10.7759/cureus.77586

**Published:** 2025-01-17

**Authors:** Feras Ayaz, Osamah Arafah, Leen Alshibi, Bayan Abu Shaar

**Affiliations:** 1 Anesthesia, King Faisal Specialist Hospital and Research Centre, Riyadh, SAU

**Keywords:** caudal block, lipid emulsion therapy, local anesthetic systemic toxicity (last), pediatric anesthesia, ventricular fibrillation

## Abstract

Local anesthetic systemic toxicity (LAST) is a rare but serious complication of regional anesthesia, particularly in neonates and infants due to their immature hepatic metabolism, reduced protein-binding capacity, and increased vascular absorption. We report the case of a two-month-old ex-premature infant who developed ventricular fibrillation following a caudal block with bupivacaine during hernia repair and circumcision. Despite negative aspiration tests, systemic absorption of bupivacaine led to toxic plasma concentrations, resulting in sudden cardiovascular collapse without preceding neurological symptoms, a presentation typical in pediatric LAST. Immediate resuscitation, including cardiopulmonary resuscitation, epinephrine, and lipid emulsion therapy, successfully restored normal cardiac rhythm. This case underscores the importance of vigilance, strict adherence to weight-based dosing, and the availability of lipid emulsion therapy in pediatric anesthesia while emphasizing prevention through careful technique, monitoring, and the use of ultrasound guidance to minimize the risk of this life-threatening complication.

## Introduction

Local anesthetic systemic toxicity (LAST) is a rare but serious complication of regional anesthesia that can lead to life-threatening central nervous system (CNS) and cardiovascular system (CVS) effects. Although the overall incidence of LAST is low, pediatric patients, particularly neonates and infants, are at significantly higher risk due to their unique physiological characteristics, including immature hepatic metabolism, reduced protein-binding capacity, and increased vascular absorption [1,2]. Infants account for a disproportionate number of LAST cases, with studies estimating an incidence of 0.76 per 1,000 regional anesthesia procedures in children [3]. The pharmacokinetics and pharmacodynamics of local anesthetics in infants, combined with their narrow therapeutic window, make this population especially vulnerable to systemic toxicity, even at dosages considered safe for older children and adults [4].

 In infants, the clinical presentation of LAST often differs from that in adults, with cardiovascular symptoms such as bradycardia, hypotension, and arrhythmias frequently appearing as initial manifestations rather than the CNS symptoms commonly observed in older patients [5]. Advances in lipid emulsion therapy have significantly improved the prognosis of LAST, but prevention remains a cornerstone of safe anesthesia practice in this high-risk population [6]. This case report and literature review provide an overview of LAST in pediatric patients and infants, covering its epidemiology, physiopathology, clinical presentations, management, and prevention, illustrated by a case of ventricular fibrillation (VF) following a caudal block in a two-month-old infant.

## Case presentation

A two-month-old ex-premature infant weighing 3 kg was scheduled for hernia repair and circumcision. Induction of anesthesia was performed using intravenous ketamine (1 mg/kg), glycopyrrolate (7 mcg/kg), propofol (3 mg/kg), and rocuronium (1 mg/kg). Mask ventilation was uneventful. However, multiple intubation attempts (two using direct laryngoscopy and one with a video laryngoscope) were unsuccessful due to the anterior positioning of the larynx, resulting in a Cormack-Lehane grade 3 view. A fourth attempt using the video laryngoscope resulted in successful intubation.

Following intubation, a caudal block was performed. The sacral hiatus was identified, and a 24G cannula was inserted. Aspiration tests were conducted before, and the injection of 3 mL of 0.25% bupivacaine yielded no cerebrospinal fluid or blood.

Shortly after the block, the ECG showed VF. The patient had no palpable pulse, prompting immediate cardiopulmonary resuscitation (CPR). Epinephrine (3 mcg) and two doses of intralipid (4.5 mL each) at two-minute intervals were administered, after which the ECG returned to a normal sinus rhythm. The total code duration was five minutes, with capnography readings consistently above 25 mmHg. Perfusion and oxygen saturation were maintained, and arterial blood gas analysis revealed mild respiratory acidosis with normal lactate levels. The procedure was completed uneventfully, and the patient was transferred to the pediatric intensive care unit while intubated.

## Discussion

LAST is a rare but potentially fatal complication of regional anesthesia, particularly in vulnerable populations such as neonates and infants. The incidence of LAST in pediatric patients has been estimated at 0.76 per 1,000 regional anesthesia procedures, with a higher prevalence in neonates and infants than in older children [1,2]. This increased risk is attributed to the unique physiological characteristics of neonates, including immature hepatic metabolism, reduced protein-binding capacity, and increased vascular absorption from injection sites [3]. Retrospective studies and registry data indicate that LAST is more likely to occur in high-risk procedures such as caudal blocks, where the richly vascularized epidural space facilitates systemic absorption of local anesthetics [4]. While the overall incidence of LAST remains low, its consequences can be severe, underscoring the need for vigilance and adherence to safe dosing practices in pediatric anesthesia.

The pathophysiology of LAST involves the systemic absorption of local anesthetics into the bloodstream, leading to toxic plasma concentrations that affect the CNS and CVS. In neonates, this risk is heightened by immature hepatic enzyme systems, particularly cytochrome P450, which metabolize amide local anesthetics such as bupivacaine and ropivacaine [5]. Additionally, neonates have lower levels of alpha-1-acid glycoprotein, the primary plasma protein that binds local anesthetics, resulting in higher free drug concentrations and an increased risk of toxicity [6]. The blood-brain barrier in neonates is also less developed, allowing greater penetration of local anesthetics into the CNS [7]. Cardiovascular toxicity, which includes arrhythmias, hypotension, and cardiac arrest, occurs due to the inhibition of sodium channels in cardiac myocytes, leading to impaired conduction and contractility [8]. The interplay of these factors makes neonates particularly susceptible to LAST even at doses considered safe for older children and adults.

The clinical presentation of LAST in pediatric patients differs significantly from that in adults. In adults, CNS symptoms such as agitation, metallic taste, tinnitus, and seizures typically precede cardiovascular collapse. However, in neonates and infants, cardiovascular symptoms are often the initial and predominant manifestations of LAST, including bradycardia, hypotension, and arrhythmias [9]. This divergence is attributed to the immature myelination of the neonatal CNS, which may delay or obscure the onset of neurological symptoms [10]. In severe cases, cardiovascular collapse can progress rapidly to cardiac arrest, as observed in our case. The absence of prodromal CNS symptoms in neonates highlights the importance of continuous monitoring for early signs of cardiovascular instability during and after regional anesthesia.

In this case, a two-month-old ex-premature infant weighing 3 kg developed VF following a caudal block with bupivacaine. Despite negative aspiration tests during the procedure, the highly vascularized caudal epidural space likely facilitated systemic absorption of the local anesthetic, leading to toxic plasma concentrations. The sudden onset of VF without preceding neurological symptoms aligns with the typical pediatric presentation of LAST. Immediate resuscitative measures, including CPR, epinephrine, and lipid emulsion therapy, were initiated, resulting in successful recovery. This case underscores the need for heightened vigilance and preparedness when performing regional anesthesia in neonates, particularly in high-risk procedures such as caudal blocks.

The management of LAST involves prompt recognition, cessation of local anesthetic administration, and initiation of supportive measures to stabilize the patient. In cases of cardiovascular collapse, as seen in our patient, advanced cardiac life support protocols should be followed, with modifications for pediatric patients. Lipid emulsion therapy has emerged as a cornerstone of LAST treatment, acting as a "lipid sink" to sequester lipophilic local anesthetics and reduce their bioavailability [11]. The American Society of Regional Anesthesia and Pain Medicine guidelines recommend an initial bolus of 1.5 mL/kg of 20% lipid emulsion, followed by an infusion of 0.25 mL/kg/min, which can be repeated as needed [12]. In this case, the administration of intralipid alongside epinephrine successfully reversed the cardiovascular collapse, consistent with the growing body of evidence supporting the efficacy of lipid therapy in pediatric LAST [13]. It is important to note, however, that high doses of epinephrine should be avoided, as they can exacerbate arrhythmias and impair the effectiveness of lipid therapy [14].

Preventing LAST in neonates and infants requires a multifaceted approach, including strict adherence to weight-based dosing guidelines, careful technique during regional anesthesia, and the use of ultrasound guidance to improve the precision of local anesthetic delivery [15]. Test doses with epinephrine and frequent aspiration during injection can help detect inadvertent intravascular placement, a common cause of LAST [16]. In neonates, where the margin of safety is narrow, even small deviations from recommended dosing can result in toxic plasma concentrations. Advances in pharmacology, such as the use of less cardiotoxic alternatives such as ropivacaine or levobupivacaine, may further reduce the risk of LAST in high-risk populations. Finally, simulation-based training in LAST management can enhance the readiness of anesthesia teams, ensuring rapid and effective responses to this rare but critical complication.

## Conclusions

LAST is a rare but serious complication of regional anesthesia, particularly in infants due to their immature physiological characteristics. Early recognition of symptoms, often cardiovascular in nature, is crucial for timely intervention in this population. While lipid emulsion therapy has improved outcomes, prevention remains key through appropriate dosing, careful monitoring, and the use of ultrasound guidance. This case highlights the importance of vigilance and tailored protocols to enhance the safety and outcomes in pediatric anesthesia.

## References

[1] Ecoffey C (2007). Pediatric regional anesthesia - update. Curr Opin Anaesthesiol.

[2] Dontukurthy S, Tobias JD (2021). Update on local anesthetic toxicity, prevention and treatment during regional anesthesia in infants and children. J Pediatr Pharmacol Ther.

[3] van den Anker JN, Schwab M, Kearns GL (2011). Developmental pharmacokinetics. Handb Exp Pharmacol.

[4] Neal JM, Barrington MJ, Fettiplace MR (2018). The Third American Society of Regional Anesthesia and Pain Medicine Practice Advisory on local anesthetic systemic toxicity: executive summary 2017. Reg Anesth Pain Med.

[5] Gitman M, Barrington MJ (2018). Local anesthetic systemic toxicity: a review of recent case reports and registries. Reg Anesth Pain Med.

[6] Weinberg G, Ripper R, Feinstein DL, Hoffman W (2003). Lipid emulsion infusion rescues dogs from bupivacaine-induced cardiac toxicity. Reg Anesth Pain Med.

[7] El-Boghdadly K, Pawa A, Chin KJ (2018). Local anesthetic systemic toxicity: current perspectives. Local Reg Anesth.

[8] Barrington MJ, Kluger R (2013). Ultrasound guidance reduces the risk of local anesthetic systemic toxicity following peripheral nerve blockade. Reg Anesth Pain Med.

[9] Zink W, Graf BM (2004). Local anesthetic myotoxicity. Reg Anesth Pain Med.

[10] Walker KJ, McGrattan K, Aas-Eng K, Smith AF (2009). Ultrasound guidance for peripheral nerve blockade. Cochrane Database Syst Rev.

[11] Weinberg GL (2010). Treatment of local anesthetic systemic toxicity (LAST). Reg Anesth Pain Med.

[12] Neal JM, Neal EJ, Weinberg GL (2021). American Society of Regional Anesthesia and Pain Medicine local anesthetic systemic toxicity checklist: 2020 version. Reg Anesth Pain Med.

[13] Shah S, Gopalakrishnan S, Apuya J, Shah S, Martin T (2009). Use of intralipid in an infant with impending cardiovascular collapse due to local anesthetic toxicity. J Anesth.

[14] Hiller DB, Gregorio GD, Ripper R (2009). Epinephrine impairs lipid resuscitation from bupivacaine overdose: a threshold effect. Anesthesiology.

[15] Di Gregorio G, Neal JM, Rosenquist RW, Weinberg GL (2010). Clinical presentation of local anesthetic systemic toxicity: a review of published cases, 1979 to 2009. Reg Anesth Pain Med.

[16] Fisher QA, Shaffner DH, Yaster M (1997). Detection of intravascular injection of regional anaesthetics in children. Can J Anaesth.

